# From the creation of the European research area in 2000 to a Mission on cancer in Europe in 2021‐lessons learned and implications

**DOI:** 10.1002/1878-0261.13632

**Published:** 2024-03-11

**Authors:** Julio Celis, Ulrik Ringborg

**Affiliations:** ^1^ Danish Cancer Society Copenhagen Denmark; ^2^ European Academy of Cancer Sciences Stockholm Sweden; ^3^ Karolinska Institutet Stockholm Sweden; ^4^ Cancer Center Karolinska Karolinska University Hospital Solna Stockholm Sweden

**Keywords:** building communities, cancer, cancer mission, ECRA, ERA, science policy, speaking with a single voice

## Abstract

In the year 2000, cancer research in Europe had the potential to make a difference as it had several unique strengths, such as a strong foundation in biomedical science, good patient registries, infrastructures that spanned from biological repositories to bioinformatic hubs as well as thriving Comprehensive Cancer Centers (CCCs) and basic/preclinical cancer research institutions of high international standing. Research, however, was fragmented and lacked coordination. As a result, Europe could not harness its potential for translating basic research discoveries into a clinical setting for the patients' benefit. What was needed was a paradigm shift in cancer research that addressed the translational research continuum. Along these lines, in 2000, European Union (EU) Commissioner Philippe Busquin established the European Research Area (ERA) and in 2002 the European Cancer Research Area (ECRA), and their political approval was a powerful catalyst for the increased involvement of scientists in science policy in the EU. In this report, we briefly describe the actions embraced by the cancer community and cancer organizations in response to Busquin's proposals that led to the creation of the EU Mission on Cancer (MoC) in Horizon 2020 in 2021.

## Graphical abstract

This report briefly describes the actions embraced by the cancer community and cancer organizations in reponse to Commissioner Busquin´s proposals in 2000 and 2002 that led to the creation of the EU Mission on Cancer.

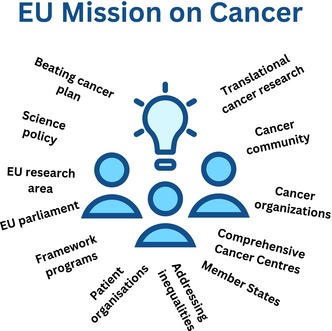

**Abbreviations**
CCCs: Comprehensive Cancer Centers
CPE: Cancer Prevention Europe
EACS: European Academy of Cancer Sciences
EC: European Commission
ECBP: Europe's Beating Cancer Plan
ECCO: European CanCer Organization
ECI: European Cancer Initiative
ECPC: European Cancer Patient Coalition
ECRA: European Cancer Research Area
EP: European Parliament
ERA: European Research Area
ERC: European Research Councill
EU: European Union
FP: Framework Program
IARC: International Agency for Research on Cancer
ISE: Initiative for Science in Europe
MoC: Mission on Cancer
OECI: Organization of European Cancer Institutes
UNESCO: United Nations Educational, Scientific and Cultural Organization

## 1 The European research area (ERA): Addressing the fragmentation of European research

The establishment of ERA, a vision championed by Commissioner Philippe Busquin in the year 2000, placed science at the core of the European knowledge society (https://cordis.europa.eu/article/id/14195‐science‐without‐frontiers‐a‐european‐research‐area). Progress in basic science was considered essential for innovation, priming scientists to accept their societal responsibility, joining forces, building, and organizing communities, and providing evidence‐based advice to inform policy. An impressive outcome of these activities by the research community was the establishment of the European Research Council (ERC) in 2007 [1, 2, 3].

Even though ERA covered all areas of science, it was clear that Europe needed to deliver the treatments and diagnostics expected by healthcare professionals and citizens. As a result, the Commissioner, with the support of the European Parliament (EP), proposed in 2002 the creation of the European Cancer Research Area (ECRA) at the ‘Towards Greater Coherence in Cancer Research’ conference (http://europa.eu/rapid/press‐release_SPEECH‐02‐408_en.htm). European cancer research was fragmented, and there was a need to create a common European strategy for cancer research. In his concluding remarks, Commissioner Busquin stated, ‘ECRA will be what you make of it’. At this point, cancer became one of the priorities in the Six Framework Program (FP6), and for the first time, an FP could explicitly support clinical research [4].

Encouraged by the outcome of the above‐mentioned conference as well as by further discussion with the cancer community and the EP, Commissioner Busquin in 2004 established a Temporary Working Group to identify ways to coordinate cancer research in Europe better and identify areas and research topics where there was a certain level of activity in the Member States that could benefit from improved coordination at the EU level. The group, chaired by M. Wim Van Velzen, a member of the EP, and composed by clinicians, basic researchers, health authorities, funders, patient organizations, and industry (Kari Alitalo, Harry Bartelink, Jose Baselga, Josep Borras, Peter Boyle, Julio Celis, Pier Paolo di Fiore, Margaret Frame, Josep Jiricny, Lucas Gianni, Les Hughes, Susan Knox, David Lane, Alex Markham, Francoise Meunier, Herbert Pinedo, Thomas Tursz, Dominique de Variola, and Otmar Wiestler) was asked to identify barriers omitting effective collaboration, as well as ways to promote development of partnership among the Member States. Following the recommendations of the Working group, the European Commission (EC) launched a call for proposals integral of FP6 in 2004 that led to the funding of the EUROCAN+Plus project in October 2005 [5], a 2‐year project coordinated by Peter Boyle, then Director of the International Agency for Research on Cancer (IARC), Lyon, France.

The project emphasized the importance of Comprehensive Cancer Centers (CCCs), which are patient‐focused entities connecting research and education with therapeutics/care and prevention and bridging research with the healthcare system. CCCs integrate every step in the cancer research continuum from basic/preclinical to early clinical, late clinical, and outcomes research for all therapeutics/care and prevention components. A European accreditation program for CCCs was developed by the Organization of European Cancer Institutes (OECI) during the presidencies of Thomas Turzs and Ulrik Ringborg and was launched in 2008 [6].

At the end of the project, the creation of a European Cancer Initiative (ECI) for translational cancer research composed of interlinked cancer centers with shared infrastructures and collaborative projects to facilitate rapid advances in knowledge and their translation into improved cancer care was proposed. Starting with a platform for translational cancer research, an ECI will be needed to coordinate basic, clinical, and epidemiological research. create informal agreements for long‐term cooperation between cancer research centers across the EU, and network funding bodies to support multinational translational research projects.

The EUROCAN+Plus project also proposed the creation of an ERA‐NET to support translational cancer research. The ERA‐NET on translational cancer research (TRANSCAN) was funded under FP7, bringing together ministries, funding agencies, and research councils having programs on translational research.

At the end of the EUROCAN+Plus project, FP6 had ended and FP7 had already started, leaving a gap that needed consideration if the translational research platform proposal was to become a reality. FPs do not allow continuity, and as a result, other options had to be explored.

In 2007, Julio Celis, with the support of the Federation of European Biochemical Societies (FEBS), founded *Molecular Oncology*, a journal that covers the fields of basic, translational, and clinical cancer research and science policy issues.

## 2 The Stockholm declaration: A paradigm shift in cancer research

Following an invitation from Ulrik Ringborg just before the end of the EUROCAN+Plus project, the directors of 16 leading European cancer research centers met in Stockholm to further define the platform for translational cancer research concept. To show their commitment, the directors signed the ‘Stockholm Declaration’, openly stating their intention to join forces, share resources and expertise, coordinate their funding, and discuss the steps towards the implementation of the declaration [7, 8].

The Stockholm Declaration signaled a paradigm shift in cancer research that was catalyzed by the dedication and collective vision of basic and clinical researchers agreeing to use a coordinated and concerted approach to work towards implementing a platform for translational cancer research by bringing together CCCs. Events leading to the publication of the Stockholm Declaration were agreed upon on November 6th, 2007, with 17 inaugural signatories.

## 3 Bridging the gap between cancer research and policy

Several informal meetings took place in 2008 to coordinate and prioritize the activities of the Stockholm group. The first steps towards making the Stockholm Declaration a reality, took place at the ‘Turning the Stockholm Declaration into Reality: Creating a World‐class Infrastructure for Cancer Research in Europe’ meeting that was held at the United Nations Educational, Scientific and Cultural Organization (UNESCO) headquarters in Paris during 2008. The conference was jointly sponsored by the Danish Cancer Society, the Initiative for Science in Europe (ISE) and the UNESCO and was attended by scientists, clinicians, policymakers, managers, patient organizations and representatives from industry. At this meeting, former Commissioner Busquin indicated that scientists must frame the challenge in such a way that it targets the specific responsibilities of the EC as separated from the national duties. Health care is a responsibility of the Member States, while research is a European competence over which the Commissioner can legislate and allocate funding. For translational cancer research the necessary integration of healthcare and research, the objective of the CCC, requires new ways of collaborations between the healthcare and the research.

**Fig. 1:**
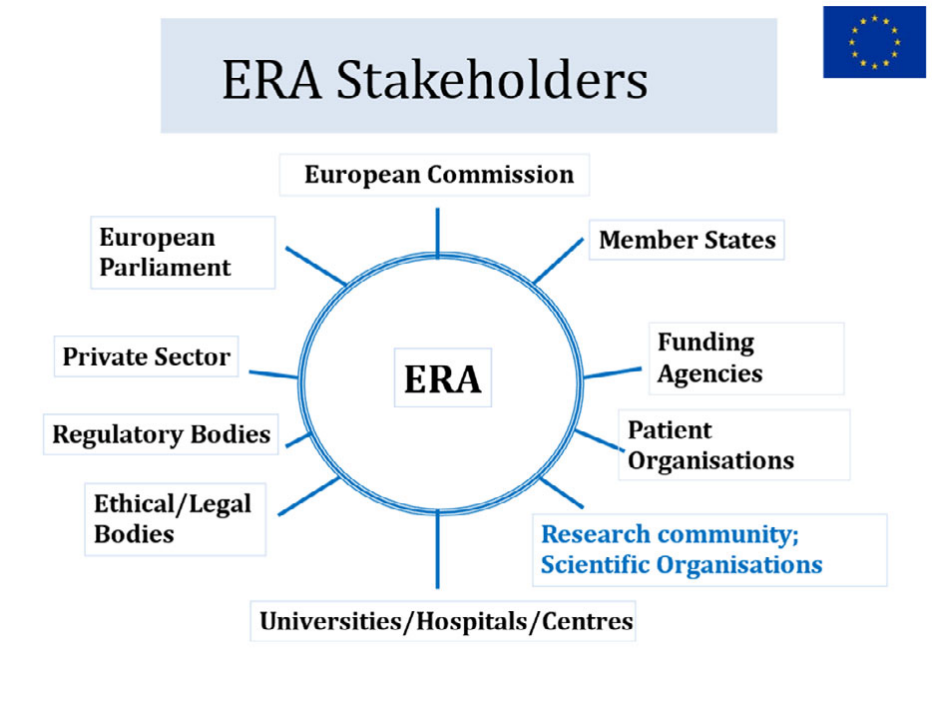
ERA stakeholders.

Commissioner Busquin suggested that it was essential to win the support of ERA stakeholders (Fig. 1), and urged the cancer community to act quickly, as financial perspectives would be discussed in 2009. Hence, the possibility of applying to FP7 to achieve continuity arose [8].

A very encouraging outcome of the meeting in Paris was that the cancer community was perceived as a well‐structured group and a reliable partner/stakeholder for planning future research activities, including setting of priorities for upcoming calls within the framework programs. At this point, connecting with FP7 was on the horizon!

At the UNESCO Conference, it was also clear that we had to bridge the gap between science and policy and the European CanCer Organization (ECCO), with its constituency of over 50 000 professionals in oncology, broad international representation, multidisciplinarity, and its prestigious bi‐annual congress, seemed uniquely positioned to channel the voice of the whole cancer community and to provide a platform for debating European cancer policy issues that may benefit the patients as well as society as a whole. As a result, Julio Celis proposed to ECCO both the creation of the Science Policy Committee in 2008, supported by well‐known policymakers and advisors (Philippe Busquin, Jose Mariano Gago, Frank Gannon, Federico Mayor Zaragoza, and Peter Lange) and the establishment of the European Academy of Cancer Sciences (EACS) at the ECCO 15 – ESMO 34 Congress in 2009. The EACS was to enhance the education of professionals working in cancer care and research by exchange, collaboration, and training of MDs and PhDs involved in biological, translational and clinical cancer research, stimulate the two‐way traffic between bench and bedside, ensure consistent authoritative scientific input in policies regarding cancer (think tank role), and promote European initiatives for translational research that would have a major impact on cancer prevention, early diagnosis and treatment. Key supporters of the Academy included Michael Baumann, Alexander M.M. Eggermont, Tim Hunt, Georg Klein, David Lane, Paul Nurse, Thomas Tursz, Umberto Veronesi, Harald zur Hausen, and Jose Mariano Gago. The first President of the EACS was Alexander Eggermont, and Julio Celis chaired the Science Policy Committee.

## 4 The EurocanPlatform network of excellence: Making the platform for translational cancer research a reality

Stemming from the EUROCAN+Plus project and the support from cancer organizations, the cancer community, and policy advisers, the EurocanPlatform Network of Excellence, led by Ulrik Ringborg, was funded by FP7 in 2011. The project engaged 23 European cancer centers, 11 Member States, and five large scientific organizations, eCancer.eu, ECCO, the European Cancer Patient Coalition (ECPC), the European Organization for Research and Treatment of Cancer (EORTC), and the Organization of European Cancer Institutes (OECI). The task was to create a translational cancer research platform to promote innovation in prevention, early detection, and therapeutics, with a focus on personalized medicine.

One of the main achievements of the EurocanPlatform was the creation of Cancer Core Europe in 2014 [9], proposed by O. Wiestler and spearheaded by A. Eggermont, who used a bottom‐up approach to initiate the process as recommended in the Stockholm Declaration. The initial centers participating in the Cancer Core were Gustave Roussy Cancer Campus Grand Paris, the Cambridge Cancer Centre, the Karolinska Institutet, the Netherlands Cancer Institute, the Vall d'Hebron Institute of Oncology and the German Cancer Research Centre with its CCC—the National Centre for Tumor Diseases. Later, the Fondazione IRCCS Istituto Nazionale dei Tumori joined the platform. Most of these centers are designated CCCs, i.e. institutions where prevention and treatment integrate with research and education.

The Cancer Core Europe's goal of linking scientific discovery from bench to bedside included the creation of a virtual ‘e‐hospital’ to enable joint research programs with a shared research infrastructure platform aimed at personalized/precision cancer medicine: (a) quality assurance and sharing of data from clinical trials; (b) molecular tumor boards for stratification of patients for clinical trials; (c) integrate diagnostic procedures including imaging; (d) expand next‐generation clinical trials to reach the necessary critical mass; anticancer agents and immunotherapy; (e) education and training; legal and ethical issues to facilitate international research collaborations.

**Fig. 2:**
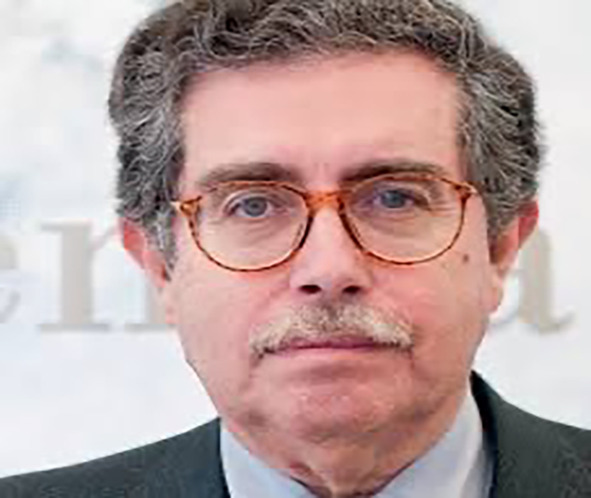
The late Jose Mariano Gago, former Portuguese Minister of Science, Technology, and Higher Education.

The vision, leadership, and support of the late Portuguese minister Jose Mariano Gago (Fig. 2) was instrumental during the various phases of the EurocanPlatform Consortium, including the creation of Cancer Core Europe, as he continuously communicated with Commissioner Moedas.

Following the initial success of Cancer Core Europe, Christopher Wild, Director of IARC, spearheaded the creation of Cancer Prevention Europe (CPE) [10]. Today, CPE is a reality composed of 10 leading European research organizations. Cancer Core Europe and CPE link the therapeutic and prevention geometries. Together, they will provide a virtual infrastructure that, in concert with other networks of cancer centers, will serve as a hub and an engine to coordinate and optimize joint cancer research efforts across Europe. It will also facilitate rapid advances in knowledge and their translation into better cancer treatment, care, and prevention. The creation of Cancer Core Europe and CPE may again initiate a discussion to establish an ECI in the future [5].

At that time, there were several other relevant initiatives in Europe aimed at accelerating the translation of research into the clinic. These included Cancer Research UK, the German Cancer Consortium, INCA in France, the Francis Crick Institute, as well as clinical infrastructures such as the European Advanced Infrastructure in Medicine (EATRIS), the European Clinical Research Network (ECRIN), and the Biobanking and Biomolecular Resources Research Infrastructure Network (BBMRI‐ERIC). In the USA, President Obama proposed the creation of a Cancer Moonshot initiative in 2016 to cure cancer along the lines of the European effort (https://www.cancer.gov/research/key‐initiatives/moonshot‐cancer‐initiative).

## 5 Bridging the cancer community efforts through horizon Europe (H2020) and beyond. Timeline of events outlining the Mission on cancer

*In 2015*, Commissioner Moedas put forward Open Innovation, Open Science and Open to the World as a new way forward for Europe and established the Research, Innovation, and Science Experts (RISE) High‐level Group to get advice on evidence and policies.

*In July 2017*, the ‘Lamy Group’ appointed by the Commission to maximize the impact of EU Research and Innovation programs suggested a mission‐oriented approach in Horizon Europe to address global challenges. Since the 3 O's were interlinked, Julio Celis and former Minister Dainius Pavalski, members of the RISE‐High‐level Group, proposed a Mission on Cancer to Commissioner Moedas based on the activities and aims of Cancer Core Europe and CPE. The mission stated that by combining innovative prevention and treatment strategies in a sustainable state‐of‐the‐art virtual European cancer center/infrastructure, it will be possible by 2030 to achieve long‐term survival of three out of four cancer patients in countries with well‐developed healthcare systems [11]. In the long term, primary prevention will modify the increasing cancer incidence. Furthermore, the proposed concerted actions will pave the way to handling the economic and social inequalities in countries with less developed systems. These efforts will also ensure that, in the long run, science‐driven and social innovations reach patients across the healthcare systems in Europe. EACS members contributed to the preparation of the proposal.

**Fig. 3:**
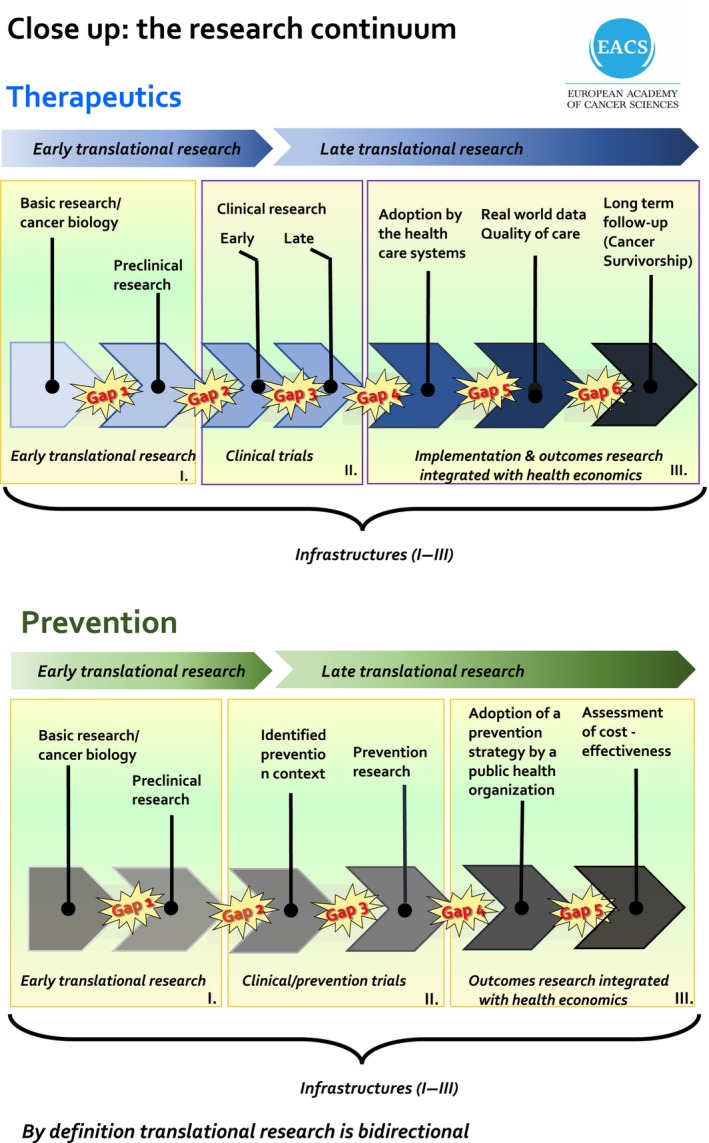
Gaps in the therapeutics and prevention cancer research continuum. Cancer research needs to be integrated, hopefully through the establishment of infrastructures for early translational research, clinical and prevention trials, and outcomes research.

**Fig. 4:**
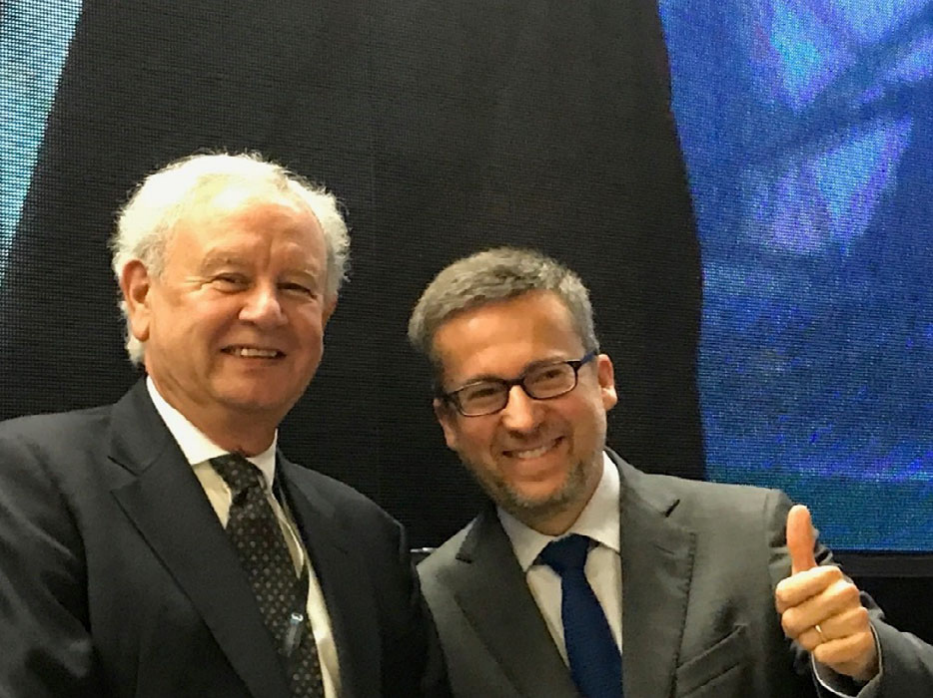
Carlos Moedas and Julio Celis at the Porto Meeting in 2018.

*February 2018*, the mission on cancer was thoroughly discussed at the first Gago Conference on ‘European Science Policy’—centered on cancer research in Europe and policy perspectives that took place at the i3S in Porto, Portugal, under the auspices of the Portuguese Agency for the Promotion of Scientific and Technological Culture, Ciência Viva, Rosalia Vargas, and with the support of the Portuguese Ministry for Science, Technology, and Higher Education Manuel Heitor (https://www.cienciaviva.pt/gagoconf/1st‐edition/). Among the issues addressed, Ulrik Ringborg, Secretary‐General of the EACS, emphasized that a Mission on Cancer must cover the entire cancer research continuum for both prevention and therapeutics (Fig. 3). The meeting also presented a unified view about establishing high‐quality, networked infrastructures to decrease cancer incidence, increase the cure rate, improve patient survival and quality of life, and ideally deal with research and care inequalities across the EU. Closing the meeting, Commissioner Carlos Moedas ratified his strong support for a mission‐oriented approach to cancer in Horizon Europe (Fig. 4).

In addition, Commissioner Moedas invited Professor Mariana Mazzucato to provide strategic recommendations to maximize the impact of the future EU Framework Program for Research and Innovation through mission‐oriented policy. Mariana Mazzucato stimulated policy debate at both national and EU levels continuously and proactively and outlined the main issues underlying the implementation of missions (https://op.europa.eu/en/publication‐detail/‐/publication/5b2811d1‐16be‐11e8‐9253‐01aa75ed71a1/language‐en).

*October 2018*, the European Competitiveness Council discussed preliminary ideas concerning missions based on a non‐paper presented by the Commission. Five potential mission areas were addressed, including curing pediatric cancer by 2030.

*November 2018*, the broad cancer mission was discussed at a Conference that took social place at the Vatican City, ‘A mission‐oriented approach to cancer in Europe: Boosting the impact of innovative cancer research’ jointly organized by the EACS and the Pontifical Academy of Sciences. The Conference concluded that as cancer is predominantly a disease of aging, focusing on the 0.6% of all cancer patients in the 0–19‐year age group will severely compromise a mission's scientific, social, and economic impact. Moreover, a mission limited to pediatric oncology would not address research in prevention and early detection, which are of fundamental importance to progress against cancer overall [12]. Pediatric oncology should be an essential component of a mission covering all cancers.

*December 2018*, it was agreed that a general cancer mission should replace the pediatric cancer mission. The debate among Member States at the level of the Competitiveness Council among Research Ministers was central to this decision.

*February 2019*, the European Competitiveness Council approved five mission areas following the Commission proposal: climate change, cancer, oceans, carbon neutrality, and food.

*June 2019*, the EACS supported Miklós Kásler, former head of the National Institute of Oncology Budapest and Minister of Human Resources in Hungary, to create the Central Eastern European Academy of Oncology, connecting 21 countries to promote cooperation in the fields of cancer prevention, treatment, and care to decrease present inequalities.

*July 2019*, Commissioner Moedas appointed Nobel laureate Harald zur Hausen, a member of the EACS, as Chair of the Horizon Europe *mission board* for *cancer* to lead the EU's research mission on cancer following a recommendation from Julio Celis.

*September 2019*, the first meeting of the Mission Board took place.

*October 2019*, the ministers responsible for research in Germany, Portugal and Slovenia signed the declaration ‘Europe: together against cancer’ (https://www.dekade‐gegen‐krebs.de/de/wir‐ueber‐uns/aktuelles‐aus‐der‐dekade/_documents/deklaration‐europa‐gemeinsam‐gegen‐krebs); and Professor Walter Ricciardi substituted Professor Harald zur Hausen due to health problems.

*October 2020*, the declaration on the need to promote cancer research in Europe, ‘Europe united against cancer’ signed on October 13, by Germany, Portugal, and Slovenia, under the Council Presidency Trio of the EU and within the scope of the German Presidency of the Council of the EU. It aimed to guide future directions for public and community support for research activities and to encourage the strengthening of collaboration with health systems across Europe.

*December 2020*, the Mission Council gave the final recommendations to the European Commission.

*February 2021*, the EC created Europe's Beating Cancer Plan (EBCP). The Cancer Plan is structured around prevention, early detection, diagnosis and treatment, and improving quality of life (https://ec.europa.eu/commission/presscorner/detail/en/ip_21_342). Through EBPC, the Commission will deal with all aspects of the disease pathway. It also plans to collaborate with the USA Cancer Mission.

*September 2021*, the MoC was launched by publishing its Implementation plan. The new goal stated, ‘By 2030, more than 3 million lives saved, living longer and better’. The mission objectives included understanding cancer, prevention and early detection, diagnosis and treatment, quality of life for patients and their families and equitable access. From 2024, the MoC “UNCAN.eu” will establish a digital platform where researchers can access and share high‐quality data to understand cancer better. EBCP, on the other hand, has launched several projects involving networking such as CraNE (organization and framework for a European network of CCCs), JANE (European networks of expertise), JARC (collective action on rare cancers), and CCI4EU (comprehensive cancer infrastructures for Europe).

MoC together with EBPC, will provide a better understanding of cancer, allow for prevention, early diagnosis, optimization of treatments, and improve cancer patients' quality of life during and beyond their cancer treatment.

*December 2023*, the EC appointed former Portuguese research minister and honorary member of the EACS Manuel Heitor to lead a group advising the EU on the interim evaluation of its ongoing research and innovation program, Horizon Europe, and plans for its successor (https://sciencebusiness.net/news/fp10/commission‐assembles‐advisers‐framework‐programme‐10).

## 6 Achievements and lessons learned

By unifying the cancer community together with main cancer organizations, the EACS has spoken with a single voice in advising the EC about (a) present unmet needs in prevention and therapeutics/care, (b) the role of translational cancer research in meeting the requirements, (c) the role of CCCs and advanced infrastructures, (d) structuring collaboration for the development of personalized/precision cancer medicine and (e) developing science policy and strategies to decrease inequalities [13, 14, 15, 16, 17].

Reaching where we stand today required building communities, identifying leaders, engaging all the relevant stakeholders along the cancer continuum, organizing science, encouraging collaboration between researchers across borders and beyond, promoting interaction and synergies at EU, national and regional levels, providing evidence‐based advice to inform policy and speaking with a single voice on wider policy issues.

## Conflict of interest

The authors declare no conflict of interest.

## Author contributions

JC and UR designed and wrote the manuscript and complemented each other with their different backgrounds and experiences.
